# Biochanin A Inhibits Ruminal Nitrogen-Metabolizing Bacteria and Alleviates the Decomposition of Amino Acids and Urea In Vitro

**DOI:** 10.3390/ani10030368

**Published:** 2020-02-25

**Authors:** Sijia Liu, Zhenyu Zhang, Samson Hailemariam, Nan Zheng, Min Wang, Shengguo Zhao, Jiaqi Wang

**Affiliations:** 1 State Key Laboratory of Animal Nutrition, Institute of Animal Sciences, Chinese Academy of Agricultural Sciences, Beijing 100193, China; liusijia1214@163.com (S.L.); zzy123779@126.com (Z.Z.); samsonet02@gmail.com (S.H.); zhengnan@caas.cn (N.Z.); 2 State Key Laboratory of Animal Nutrition, The Institute of Subtropical Agriculture, The Chinese Academy of Sciences, Changsha 410125, Hunan, China; mwang@isa.ac.cn

**Keywords:** biochanin A, rumen, urea, amino acid, bacteria composition

## Abstract

**Simple Summary:**

Plant bioactive compounds have been chosen as alternative antibiotic to promote animal productivity. Biochanin A is a type of naturally occurring bioactive compound. It is O-methylated isoflavone and is found in red clover, alfalfa sprouts, and other legumes. The aim of this study was to determine the effect of biochanin A on rumen microbial fermentation and composition. The results show that biochanin a increases microbial gas production, but has no effect on volatile fatty acid (VFA) production. Microbial urease activity was inhibited by Biochanin A with the IC50 of 320 nM. Biochanin A also inhibited the degradation rate of Val, Lys, Met, Leu and total amino acids, respectively. The inhibition of urease activity and amino acid decomposition by biochanin A resulted in a reduction in ammonia. The 16S rRNA gene sequencing showed that biochanin A reduced the abundance of proteolytic bacteria *Prevotella* and *Streptococcus*. Therefore, biochanin A reduced the production of ammonia by inhibiting proteolytic bacteria and its decomposition of urea and amino acids activity.

**Abstract:**

Biochanin A is a naturally occurring flavonoid compound that is found in plant species such as red clover (*Trifolium pretense*) and alfalfa (*Medicago sativa*). Flavonoids have been reported to regulate ruminal fermentation, and the objective of this study was to evaluate the effects of biochanin A on ruminal microbial composition and nitrogen metabolism. The experiment was performed by in vitro batch culturing of a control (without biochanin A) and a biochanin A treatment. Following a 24-h incubation, gas production and the amounts of ammonia-nitrogen (NH_3_-N), volatile fatty acid (VFA), and amino acids were measured. Microbial population using 16S rRNA gene sequence. We found that the addition of biochanin A significantly increased microbial gas production; but had no effect on VFA production. Biochanin A supplementation also resulted in reduced microbial urease activity with half the maximal inhibitory concentration of 320 nM and also inhibited the degradation rates of total amino acids, valine, lysine, methionine and leucine by 18%, 56%, 37%, 13%, and 12%, respectively. This inhibition of urease activity and amino acid decomposition resulted in a significant reduction in the NH_3_-N concentration. High-throughput sequencing of the 16S rRNA sequence to monitor microbial composition showed that biochanin A significantly reduced the abundance of the proteolytic bacteria *Prevotella* and ureolytic bacteria *Selenomonas*, but increased the abundance of the lactic acid metabolizing bacteria *Veillonella* and *Megasphaera*. In conclusion, biochanin A reduced the production of ammonia by inhibiting proteolytic bacteria and their decomposition of urea and amino acids.

## 1. Introduction

The inclusion of antibiotics, such as monensin, as a feed additive has been reported to inhibit hyper ammonia-producing bacteria, which leads to slower ammonia production and the increased ruminal bypass of proteins [1,2]. However, a drawback to the use of antibiotics as growth-promoters in animal feed is that it can promote the emergence of antibiotic-resistant bacterial strains [3]. Recently, flavonoids and other phenolic compounds have been studied and found to regulate ruminal microbial fermentation [4,5]. Flavonoids are the products of plant secondary metabolism, and their biological activities depend on structural characteristics, such as their pattern of glycosylation. In ruminant production, different flavonoids can have different antimicrobial effects which influence the extent to which rumen fermentation can be altered [4,6]. Biochanin A is a natural organic O-methylated isoflavone found in the pasture legume red clover (*Trifolium pratense*) and alfalfa *(Medicago sativa)* [7]. Biochanin A improves ruminal fiber degradation by altering the composition of cellulolytic bacteria 6], and has been recognized as an effective antibiotic alternative for mitigating sub-acute ruminal acidosis by inhibiting the growth of amylolytic bacteria [8].

The rumen has a metabolically diverse microbial community that mediates the first enzymatic steps in the digestion of dietary components [9]. This is essential for the degradation and utilization of proteinaceous or non-proteinaceous nitrogen. Proteolytic microbes in the rumen produce proteases and peptidase, which convert proteins into peptides and amino acids [10]. Hydrolyzed oligo-peptides and amino acids can be transported to microbial cells to synthesize microbial proteins or be deaminated to ammonia, which can be assimilated by microorganisms [9,10]. Urea, a type of non-proteinaceous nitrogen, is included as a common supplement in the diets of ruminants in order to compose microbial crude protein (MCP), and reduce the cost of animal feed [11,12]. However, the rate of urea hydrolysis to ammonia often exceeds the rate of ammonia utilization, which results in the low efficiency of urea-N utilization and microbial protein synthesis [12,13]. Therefore, the rapid production of ammonia from proteins or urea from the diet leads to low-efficiency rumen fermentation, excess emission of nitrogen to the environment and decreased milk protein production in dairy cows. Biochanin A has in vitro antimicrobial activity against the pure culture hyper ammonia-producing bacteria *Acetoanaerobium sticklandii* [14] and *Peptostreptococcus anaerobius* [15], leading to reduced deamination in the rumen. In addition, biochanin A also inhibited the pathogens *C. clostridioforme* and *C. perfringens*, suggesting that it can play a role in improving gut health [16]. We hypothesized that biochanin A could alter the microbial composition of the rumen as well as nitrogen metabolism. To test this, the objective of this study was to determine the effect of biochanin A on rumen microbial composition and decomposition of amino acids and urea using in vitro batch culturing.

## 2. Materials and Methods

The experimental procedures involving the care and management of dairy cows were approved by the Animal Care and Use Committee for Livestock of the Institute of Animal Sciences, Chinese Academy of Agricultural Sciences (No. IAS201914).

### 2.1. Preparation of the Rumen Microbial Mixture

Rumen fluid was sampled from three lactating Holstein dairy cows (550 ± 23 kg body weight) before morning feeding. All cows were fed a Total Mixture Ration (TMR) diet consisting of 17.3% alfalfa hay, 18.7% corn silage, 11.3% soybean meal, 4.2% rapeseed meal, 2.1% cotton aphid, 2.1% puffed soybeans, 4.2% beet pulp, 10.4% whole cottonseed, 25.6% corn, 0.5% salt and 3.6% premix. The rumen fluid was filtered through six layers of cheesecloth, allowing the separation of solids from the liquid fraction. One part of the liquid fraction was dispensed into centrifuge tubes, while another was transferred using a syringe into serum bottles containing an anaerobic storage solution, as described in the ‘anaerobic medium preparation’ paragraph in a 1:1 (vol : vol) ratio to create a rumen microbial inoculum. All rumen fluid samples were frozen on dry ice, transported to the laboratory and stored at −80 °C until further use.

### 2.2. Anaerobic Medium Preparation

The ruminal fluid samples from centrifuge tubes were thawed in water (room temperature) and centrifuged (13,000× *g* at 4 °C for 5 min) and the supernatants were collected as clarified rumen fluid samples. One hundred mL of the anaerobic medium contained 10 mL clarified rumen fluid, 0.05 g starch, 0.05 g glucose, 0.05 g cellobiose, 0.05 g amino acid mixture, 0.6 g NaHCO_3_, 0.31 mL volatile fatty acid (VFA) solution, 50 mL inorganic salt solution, 0.1mL trace element solution, 0.05 g hydrochloric acid cysteine salt, 0.1 mL heme pigment (0.5 mg/mL), and 0.1 mg resazurin. The inorganic salt solution contained (per liter) 0.2 g CaCl_2_, 0.2 g MgSO_4_, 1.0 g K_2_HPO_4_, 10.0 g NaHCO_3_ and 2 g NaCl. The composition of 1 L of the trace element solution was 300 mg H_3_BO_3_, 100 mg ZnSO_4_∙7H_2_O, 30 mg MnCl_2_∙4H_2_O, 20 mg CoCl_2_∙6H_2_O, 30 mg Na_2_MoO_4_∙2H_2_O, 10 mg Na_2_SeO_3_, 20 mg NiCl_2_, 10 mg CuCl_2_∙2H_2_O and 150 mg FeCl_2_∙4H_2_O. The VFA solution contained 17 mL acetic acid, 6 mL propionic, 4 mL n-butyric, and 1 mL each of n-valeric, isovaleric, isobutyric and 2-methylbutyric acid. The anaerobic medium was prepared under a continuous flow of CO_2_ for 3 h, and adjusted pH to 6.8. The medium was transferred to an anaerobic chamber (Plas-Labs, MI, USA) containing 9.95% H_2_, 9.99% CO_2_ and 80.06% N_2_, distributed into Hungate tubes (10 mL per tube) sealed with rubber stoppers, and then autoclaved at 125 °C for 15 min. The anaerobic storage solution was prepared by making 30% glycerol solution using anaerobic medium as dilution. It was deoxidized under a continuous flow of CO_2_, distributed into serum bottle in anaerobic chamber, and autoclaved as described above.

### 2.3. In Vitro Batch Fermentation and Sampling

The experiment consisted of a control (without biochanin A) and a biochanin A treatment (final concentration of 0.03 mg/mL) [17] and was conducted in triplicate. Aliquots (200 μL) of the rumen microbial inoculum were mixed with 50 μL of biochanin A (Sigma-Aldrich) solution (6 mg/mL dissolved in dimethyl sulfoxide (DMSO) and passed through a 0.22 μm filter) or with 50 μL DMSO solvent alone, and inoculated into each anaerobic medium-containing tube. All adjustments were performed in the anaerobic chamber. Six inoculated anaerobic culture tubes were placed in an incubator and cultured at 39 °C for 24 h as the first generation. A total of 200 μL of culture from the first generation and 50 μL of biochanin A solution (6 mg/mL) or DMSO solvent were transferred by inoculation to each new tube with the anaerobic medium incubated as the second generation at 39 °C for 24 h. The transfer process was continued until the fourth generation. The fermentation liquid was collected after 24 h of incubation for each generation, and used for NH_3_-N measurements, VFA detection and microbial DNA extraction. The gas pressure in each tube was recorded after 24 h of incubation for each generation using a digital pressure gauge (ConST, Beijing, China) and converted to a gas volume value using the following equation: GP = P × V / 101.3, where GP is the gas production (mL), P is the pressure (kPa), V is the volume of the gas in Hungate tube (ml), and 101.3 is the standard Atmospheric pressure (kPa). At the fourth generation, the culture was sampled at 12 and 24 h for use in the amino acid concentration detection assay.

### 2.4. Inhibition of Urease Activity

Urease activity was determined by measuring the amount of ammonia released from urea. Ruminal fluid samples (2 mL) from centrifuge tubes were thawed in water (room temperature) and centrifuged (12,000× *g*, 10 min, 4 °C) and the pellet was washed twice with 1 mL 50 mM 2-[4-(2-hydroxyethyl)piperazin-1-yl]ethanesulfonic acid (HEPES) buffer (pH = 7.5). The microbial cells were lysed by sonication on ice (300 W for 5 min using 5 s on and 5 s off cycles) and centrifuged at 12,000× *g* for 10 min at 4 °C. The supernatants were collected as microbial crude enzyme solutions for urease activity determination. Biochanin A was added to the supernatants in different concentrations (0, 0.005, 0.01, 0.03, 0.1, 0.2 mg/mL). Each concentration was analyzed in triplicate.

The protein content of the crude enzyme solution was determined using a Bradford Kit (Amresco, Beijing, China). The assay solution consisted of the crude enzyme solution (0.5 mL) and urea (0.5 mL, 50 mM stock). The reaction was left at 37 °C for 20 min, before the concentration of the produced ammonia was determined as previously described, using the method based on the Berthelot (phenol-hypochlorite) reaction [18]. One unit of urease activity was defined as the amount of enzyme required to release one nmol ammonia per min per mg of crude protein.

### 2.5. 16S rRNA Gene Sequencing

Genomic DNA was extracted from microbial cell pellets, derived from four generations of cultures, using cetyltrimethylammonium bromide (CTAB) and the bead beating method [18]. Briefly, samples were homogenized using 0.5 g zirconium beads (0.5 mm diameter) in 0.8 mL CTAB buffer (100 mM Tris(hydroxymethyl)aminomethane (Tris-HCl), pH 8.0; 1.4 M NaCl; 20 mM Ethylenediaminetetraacetic acid (EDTA); 2% Hexadecyl trimethyl ammonium Bromide (CTAB)) on a Mixer Mill MM 400 (Retsch, Haan, Germany) for 60 s at 30 Hz. Phenol:chloroform:isoamyl alcohol (volume 25:24:1) was used to facilitate the removal of proteins and impurities and isopropanol was used for precipitating the DNA. The extracted DNA was assessed by agarose gel electrophoresis and quantified using a Nanodrop spectrometer (Thermo Scientific, USA).

The V3-V4 region of the 16S rRNA gene was amplified by Polymerase Chain Reaction (PCR) using the primers 338F (5′-ACTCCTACGGGAGGCAGCAG-3′) and 806R (5′-GGACTACHVGGGTWTCTAAT-3′). The reactions were performed in a MyCycler Thermal Cycler (Bio-Rad, USA) in a 50-μL reaction volume containing 5 μL of 10 × PCR buffer (Invitrogen, Carlsbad, CA, USA), 1 μL of dNTP mixture (10 mM), 1.5 μL of each forward and reverse primer (10 mM), 0.4 μL of Platinum Taq DNA polymerase (Invitrogen), 2 μL of microbial DNA (100 ng mL^−1^), and 38.6 μL of sterile ddH_2_O. Polymerase Chain Reaction amplification began with a 5 min denaturing step at 95 °C, followed by 30 cycles of 95 °C for 30 s, 58 °C for 30 s, and 72 °C for 1 min; extension was achieved at 72 °C for 15 min. Polymerase Chain Reaction amplicons were sequenced at Majorbio (Shanghai, China) on an Illumina MiSeq according to standard protocols.

### 2.6. Sequencing Data Analysis

Sequences were analyzed using the QIIME package [19] in our lab. The raw reads from Illumina sequencing were trimmed to remove sequencing adaptors and noise and then filtered to remove reads with ambiguous bases. Low-quality raw reads were eliminated using Trimmomatic [19]. Paired-end reads were merged using FLASH [20] with the following parameters overlap >10 bp and a mismatch rate <20%. Chimera sequences were detected and removed using the UCHIME denovo algorithm [21]. Operational taxonomic units (OTU) were clustered at a cut-off value of 0.97 similarity using USEARCH from the QIIME package [22,23]. The OTUs were assigned to taxa by the RDP classifier based on the Greengenes database 13_5 [21]. The OTUs were filtered based on their count (OTU <5). All the sequences were submitted to the NCBI Sequence Read Archive (SRA; http://www.ncbi.nlm.nih.gov/Traces/sra/), under accession number SRA: SRP222908.

### 2.7. Microbial Fermentation Analysis

Samples collected from four generations of the cultures were centrifuged (12,000× *g* at 4 °C for 10 min), and the supernatants were mixed with 25% metaphosphoric acid. VFAs were determined by gas chromatography (Agilent 7890A, Wilmington, USA). The NH_3_-N concentration was determined using a Berthelot ammonia assay kit (Jiancheng, Nanjing, China). 

Amino acid content was determined using the sulfosalicylic acid method. Samples of 800 μL of fourth generation cultures were collected and mixed with 200 μL of sulfosalicylic acid (20%), After centrifugation at 12,000× *g* for 20 min at 4 °C, the supernatants were passed through a 0.22 μm filter. An amino acid analyzer (Hitachi L-8900, Tokoy, Japan) was used for amino acid detection. The amino acid degradation rate was calculated using the following equation: DR (%) = 100% × (c_t_ - c_0_) / c_0_, where DR is the degradation rate, “c_t_” is amino acid concentration at time t, “c_0_” is amino acid concentration at time 0 h.

### 2.8. Statistical Analysis 

The differences between biochanin A treatment and control for rumen NH_3_-N, amino acid degradation rate, gas production and VFAs in each generation or time were analyzed using ANOVA procedure of SAS (SAS Institute Inc., Cary, NC, USA). The biochanin A treatment and control were the fixed effect. Bacterial relative abundance data were analyzed for differential significance using the Kruskal–Wallis test in SAS. The difference in beta diversity in each generation for the different treatments was determined using ANOSIM in QIIME. *p* < 0.05 indicates a significant difference, *p* > 0.05 indicates no significant difference.

## 3. Results

### 3.1. Changes in Levels of Amino Acids and Ammonia

**Figure 1:**
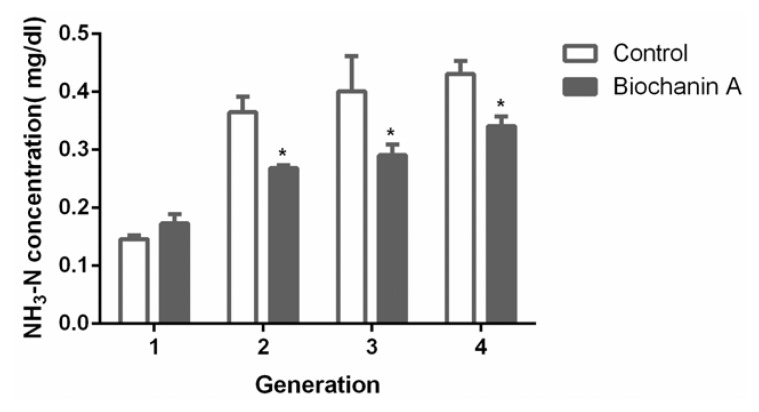
The effect of biochanin A on NH_3_-N concentration in an anaerobic culture. Treatments included controls (only dimethyl sulfoxide (DMSO)) and anaerobic medium with exogenous addition of biochanin A. Asterisks (*) indicate a significant difference between treatments (*p* < 0.05). Error bars represent SEM.

We observed that NH_3_-N production increased with successive generations. There was no significant effect on NH_3_-N level at the initial addition of biochanin A (*p* > 0.05); however, by the second, third and fourth generations, biochanin A inhibited NH_3_-N production by 26.4%, 28.0% and 20.9% respectively (*p* < 0.05) (Figure 1). 

**Figure 2:**
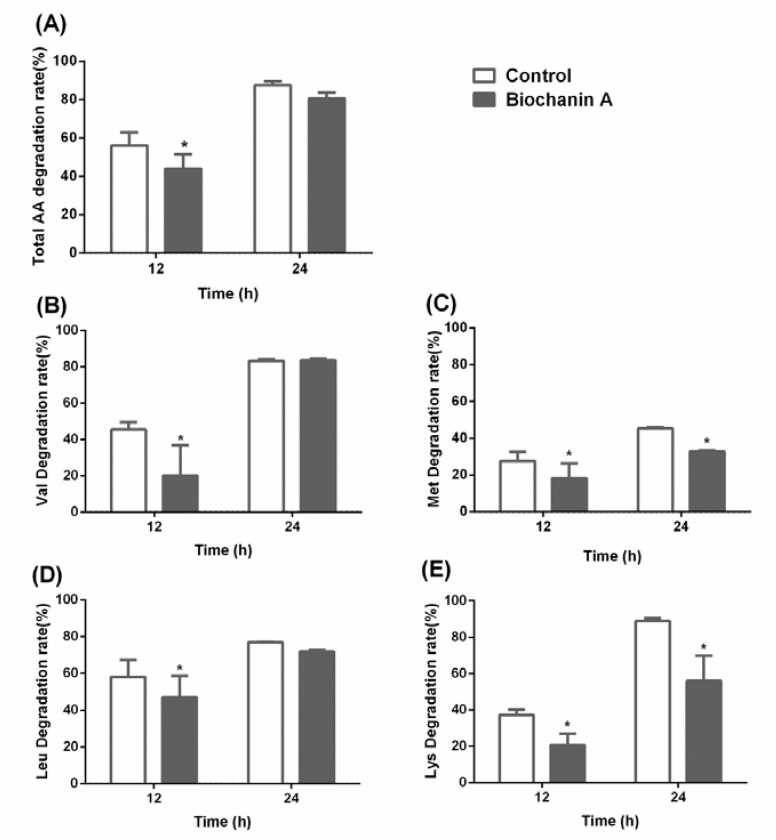
The effect of biochanin A on amino acid degradation rate in culture generation 4. The degradation of total amino acids (AA) (**A**), valine (Val) (**B**), methionine (Met) (**C**), leucine (Leu) (**D**), lysine (Lys) (**E**) during culture incubation. Treatments included controls (only DMSO) and anaerobic medium with exogenous addition of biochanin A. Asterisks (*) indicate a significant difference between treatments (*p* < 0.05). Error bars represent SEM.

In the fourth generation, 15 amino acids were detected, and their degradation rates were calculated. Compared to the control group, the biochanin A group showed a significantly lower degradation of valine (Val), methionine (Met), leucine (Leu) and lysine (Lys) (*p* < 0.05). The degradation rates of Val, Met, Leu and Lys, as well as total amino acids, were inhibited by 55.84%, 37.03%, 12.63%, 11.74% and 18.00%, respectively, compared to the control with no biochanin A (Figure 2).

**Figure 3:**
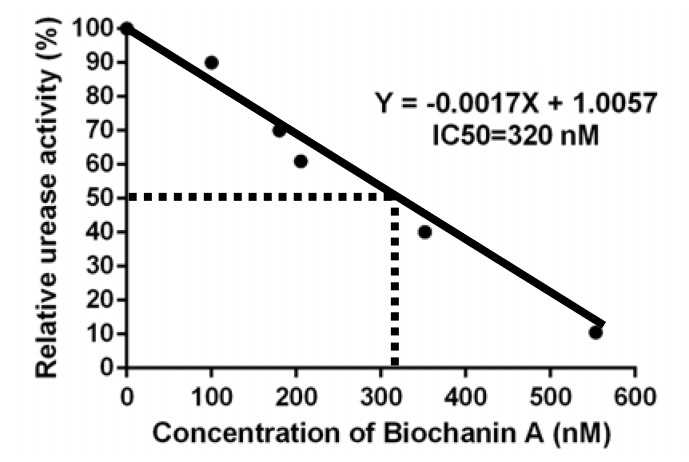
Biochanin A inhibits rumen bacterial urease activity.

Biochanin A inhibited urease activity in crude enzyme extracts from the rumen fluid of dairy cows in vitro, with a half maximal inhibitory concentration (IC50) of 320 nM (Figure 3).

### 3.2. Gas Production and VFA Levels

**Figure 4:**
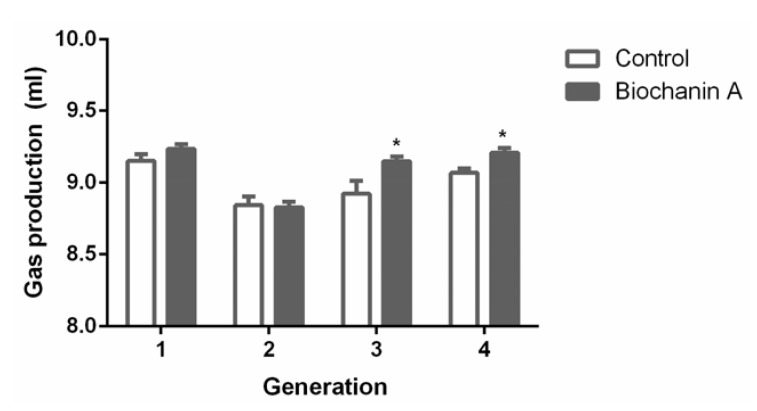
The effect of biochanin A on gas production in anaerobic medium. Treatments included controls (only DMSO) and anaerobic medium with exogenous addition of biochanin A. Asterisks (*) indicate a significant difference between treatments (*p* < 0.05). Error bars represent SEM.

**Figure 5:**
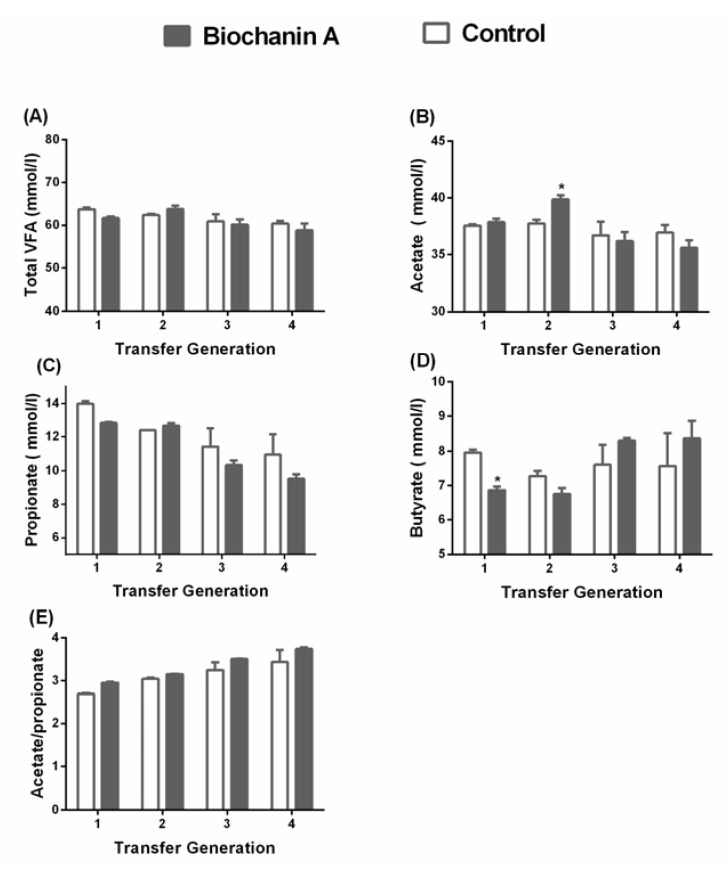
The effect of biochanin A on volatile fatty acid (VFA) production in anaerobic medium. Treatments included controls (only DMSO) and anaerobic medium with exogenous addition of biochanin A (*p* > 0.05). Error bars represent SEM.

Biochanin A supplementation increased gas production by 2.5% and 1.5% in the third and fourth generations, respectively (*p* < 0.05) (Figure 4). As shown in Figure 4, there were no significant differences in total VFAs and propionate levels between the treated and control cultures (*p* > 0.05) (Figure 5). The acetate:propionate ratio between the treated and control cultures were not significantly different (*p* > 0.05). The acetate and butyrate levels were not significantly (*p* > 0.05) changed by biochanin A at each time, except for 2 h and 1 h, respectively.

### 3.3. Change in Abundance of Ruminal Bacteria 

In total, 1,506,045 sequence reads were obtained from the 40 samples. The total number of reads from each sample varied from 29,503 to 45,507 and the average number of reads was 37,651. After removing chimeric sequences, the remaining 1,506,045 sequences were used to generate OTUs. All sequences were assigned to 1398 OTUs using a cut-off of 97% similarity, and 1322 filtered OTUs were kept for the taxonomy and relative abundance analysis.

**Figure 6:**
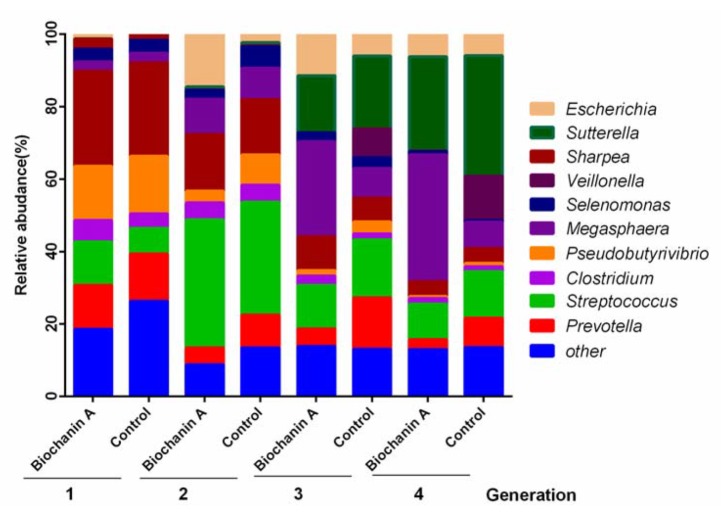
The effect of biochanin A on the composition of the predominant bacterial genera in anaerobic media of different culture generations. Treatments included controls (only DMSO) and anaerobic medium with exogenous addition of biochanin A.

**Figure 7:**
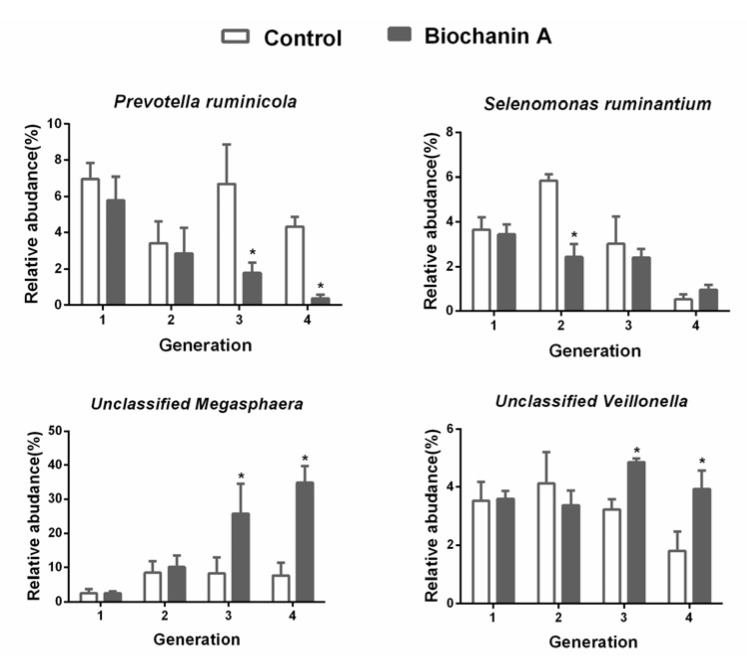
The effect of biochanin A on bacterial abundance in anaerobic media of different culture generations. Treatments included controls (only DMSO) and anaerobic medium with exogenous addition of biochanin A. Asterisks (*) indicate a significant difference between treatments (*p* < 0.05). Error bars represent SEM.

Collectively, 14 bacterial phyla were identified, of which *Firmicutes, Bacteroidetes,* and *Proteobacteria* were predominant. The five predominant genera included *Megasphaera* (average of 28%), *Clostridium* (average of 28%), *Prevotella* (average of 12%), *Escherichia* (average of 12%), and *Veillonella* (average of 12%) (Figure 6). Biochanin A supplementation caused a significant decrease in the abundance of the proteolytic bacteria *Prevotella ruminicola* and the ureolytic bacterium *Selenomonas ruminatium,* but an increase in the lactic acid metabolizing bacteria unclassified *Veillonella* and *Megasphaera* (*p* < 0.05) (Figure 7). 

**Figure 8:**
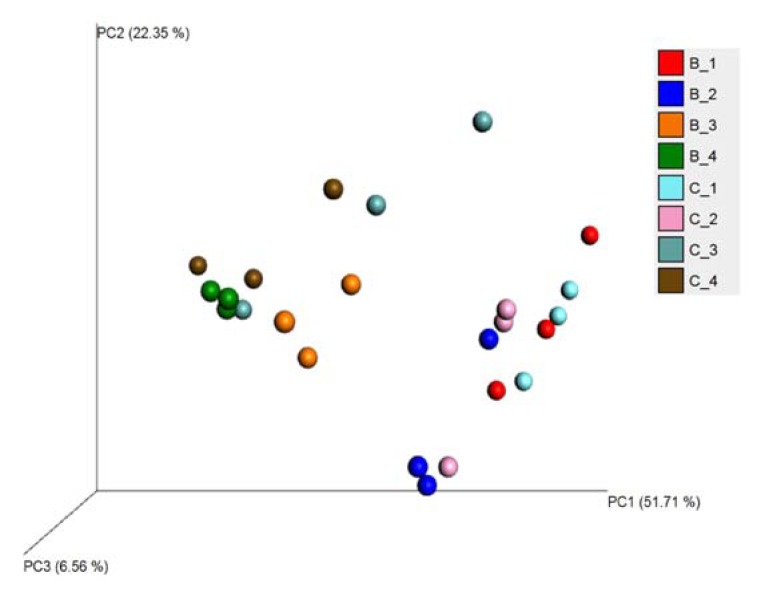
Principal coordinate analysis (PCoA) showing the diversity among all samples. In the legend, B represents biochanin A samples, C represents the control group, and the digital represent the culture generation.

Beta diversity showed that the composition of the microbial community varied between generations (Figure 8). There was no significant difference between biochanin A and the control during the first and second generations (*p* > 0.05), while a difference was seen in the third and fourth generations (*p* < 0.05).

## 4. Discussion

In this study, we found that NH_3_-N production was significantly decreased in cultures with biochanin A supplementation. Harlow et al., (2016) [7] also found that biochanin A reduced ammonia production in the rumen and increased the average daily gains of steers. Dietary protein and urea are the substrates for ammonia production in the rumen, and the suppressed ammonia outbreak has the benefit for increasing ruminal microbial protein synthesis efficiency and by-pass protein mass, which help to provide more metabolizable protein for ruminants [24]. We also found that gas production was increased during fermentation with biochanin A supplementation. Generally, the gas production is an indicator of efficiency of ruminal microbial fermentation, and more gas production indicates the high efficiency of nutrient degradation and utilization by microbes [25,26]. However, we did not observe a large difference in VFA production, which is not consistent with a previous study [6] which reported that biochanin A improved fiber degradation and VFA production by changing cellulolytic bacteria competition and guild composition [6]. The potential reason was that the medium we used was cellobiose in the medium, which can support the growth of most cellulytic bacteria, but the one they used contained ground hay—the type of substrate we used may be one reason for its inconsistency with other studies.

Dietary protein or amino acids are an important source of ammonia, and amino acid deamination is a rapid and key step for ammonia production [27]. Low degradation of amino acids leads to a decrease in ammonia production. Finally, we found that the total amino acid degradation rate and that of some individual amino acid including valine, lysine, methionine and leucine significantly decreased when cultures were treated with biochanin A. Valine and leucine belong to branch chain amino acids, which have been verified to have the function of milk protein synthesis regulation by the active cellular mTOR signal pathway in mammary glands as signaling molecules [28,29]. Lysine and methionine are limiting amino acids in dairy cow’s diets, and the supplementation of these two amino acids in metabolizable protein improves nitrogen efficiency and milk protein production [30]. Accordingly, the lower degradation of branch chain or limiting amino acids induced by biochanin A is helpful for the nitrogen metabolism and milk production.

Urea is used as a non-protein nitrogen resource in diets; however, it is rapidly degraded to ammonia by urease produced from rumen bacteria [13,31]. In this study, we found that biochanin A can effectively inhibit rumen urease activity and the following urea degradation, which thereby reduces rumen ammonia production. Biochanin A had the IC50 of 320 nM for urease activity. Previous studies revealed that acetohydroxamic acid was a standard and industrially applied ruminal urease inhibitor, and had IC50 of 5340 nM which is 16.7-fold higher compared to biochanin A [32], which indicates that biochanin A was a novel and highly efficient urease inhibitor. The slow release of ammonia enhances ammonia integration carbon compounds produced via sugar metabolism, leading to the synthesis of amino acids required for microbial protein synthesis [33]. Therefore, controlling the decomposition rate of urea to ammonia can improve the utilization efficiency of urea-N to microbial protein [4].

The 16S rRNA gene sequencing revealed that biochanin A supplementation induced the changes in the ruminal bacterial composition and its effects also depended on the generation. Biochanin A significantly increased the abundance of the proteolytic bacterium *Prevotella ruminicola*, which was the predominant bacteria in the rumen. *P. ruminicola* synthesizes proteases, peptidases and deaminases to generate ammonia from dietary protein. This could result from exposure to O_2_. The medium was not thermally degassed, so the small amount of molecular oxygen could select for O_2_-tolerant species, such as Proteobacteria, which often produce catalase and superoxide dismutase. Flythe et al., (2010) [9] found that biochanin A inhibited the growth of the hyper ammonia-producing bacterium *Acetoanaerobium sticklandii*, but no data were reported for *Prevotella*. The decreased abundance of *Prevotella* in this study could have contributed a lot to the low levels of degradation of amino acids and production of ammonia. However, we did not observe the representative hyper ammonia-producing bacteria *Acetoanaerobium sticklandii* and *Peptostreptococcus anaerobius* and significant changes in *Clostridum aminophilum* [34,35], which may be due to the low taxonomy resolution ratio of the short 16S rRNA gene sequenced here. We also found that the abundance of ureolytic bacterium *Selenomonas ruminatium* was significantly decreased by biochanin A in generation two. The combination of the inhibition of urease-producing bacteria and enzyme activity had a stronger contribution to reducing urea degradation to ammonia. This is the first report, as far as we know, that shows that biochanin A has the ability to inhibit ureolytic bacteria growth and their urease activity, but the mechanism is still unknown.

Here, we also found that biochanin A promoted the abundance of the lactate-utilizing bacteria *Megasphaera* and *Veillonella*. Harlow et al., (2017) [18] observed that biochanin A inhibited the amylolytic or lactate-producing bacteria *Streptococcus bovis*, *Lactobacillus reuteri*, and *Selenemonas ruminatium*, and increased the dry matter digestibility of hay with corn starch. The balance of lactate producing and utilization is very important for rumen microbial fermentation. The accumulation of lactate easily leads to ruminal acidosis, which is a common digestive disorder in rumen, and this has negative effects on both health and production performance for ruminants [36]. Starch is an important substrate of lactate-producing bacteria, so a diet with a large proportion of starch has a large risk of ruminal acidosis in a ruminant’s feeding system. Harlow et al., (2017) [8] found that biochanin A mitigated sub-acute ruminal acidosis by inhibiting starch-utilizing bacteria. Here, we found lactate-utilizing, not -producing, bacteria were promoted by biochanin A, indicating improvement of ruminal acidosis.

## 5. Conclusions

Biochanin A reduced the abundance of proteolytic bacteria, and resulted in the inhibition of urease activity and the rate of amino acid decomposition to ammonia in the rumen. Biochanin A also promotes the abundance of lactate-utilizing bacteria to improve ruminal acidosis. The beneficial effects of biochanin A on rumen fermentation may include more efficient nitrogen utilization and animal production, highlighting its promise as a feed additive for ruminants.
